# Highly esteemed science: An analysis of attitudes towards and perceived attributes of science in letters to the editor in two Dutch newspapers

**DOI:** 10.1177/0963662519878988

**Published:** 2019-10-08

**Authors:** Stefan P.L. de Jong, Elena Ketting, Leonie van Drooge

**Affiliations:** Leiden University, The Netherlands; Luris, The Netherlands; Inspectorate SZW, The Netherlands; Rathenau Instituut, The Netherlands

**Keywords:** academic autonomy, attitudes towards science, attributes of science, conflicts of interest, content analysis, letters to the editor, organization of science

## Abstract

Understanding attitudes towards science is crucial to safeguard the future of science, the application of its results and the inclusivity of decision-making processes related to science and technology. Most studies focus on attributes of social groups to explain attitudes towards science. In this study, we aim to move the discussion forward by focusing on perceived attributes of science itself by analysing over 300 letters to the editor in two Dutch national newspapers. The authors of these letters express a large degree of trust in science as a source of societal progress, if research is conducted according to a specific set of rules. Yet, they believe that these rules are under attack. The interests of universities as organizations and individual academics as well as the involvement of industry and government in research are perceived as conflicting with these rules. We conclude with recommendations for further research and practice.

## 1. Introduction

This article explores attitudes towards science in letters to the editor in two Dutch national newspapers. It aims to move beyond the identification of attributes of certain social groups that affect their attitude, and towards the identification of perceived attributes of science that might affect attitudes.

There are four main reasons for the interest in attitudes towards science. First, positive attitudes towards science are seen as a requirement for students to aspire a scientific career (George, 2006; Osborne et al., 2003). Second, the prevalence of positive attitudes seems to be conducive to scientific results being used in an increasingly information- and technology-driven society; most notably in evidence-based policymaking and as a motor of the knowledge economy (Allum et al., 2008; Pechar et al., 2018). Third, investments in science are thought to be secured by a general positive attitude towards science (Knight and Barnett, 2010). Finally, positive attitudes would allow the public to engage in decision-making processes related to science and technology (Lee and Kim, 2018; Sturgis and Allum, 2004). All these reasons imply that science and society cannot do without each other.

Attitudes of the general public (e.g. Allum et al., 2008; Bauer et al., 1994; Price and Lee, 2013) as well as of specific groups have received most of the attention. These particular groups can be related to the four reasons for the interest in attitudes towards science. Related to the first reason is the study of attitudes of students and to a lesser extent teachers (Michaluk et al., 2018; Yager and Yager, 1985). The other three reasons may explain the attention for groups that are believed to hold less positive attitudes towards science, such as the religious (Allum et al., 2014; DiMaggio et al., 2018; McPhetres and Zuckerman, 2018); conservatives (Hmielowski et al., 2014; Motta, 2018) and women (Hayes and Tariq, 2000; Jones et al., 2000). Remarkably, despite scientists being as much members of society as any other group, their attitudes only have received limited attention (Albert et al., 2008).

Surveys are the dominant method to study this topic. Their advantage is their ability to evidence generalizable relationships, or the lack thereof, between attitudes towards science and educational background, gender or scientific knowledge, for instance (Nisbet and Goidel, 2007). Also, surveys can be standardized, which facilitates comparison over time and space. The gathered information, however, is based on questions manufactured by researchers. Potentially important and newly arising dimensions comprising attitudes towards science might therefore remain unexposed.

Other approaches to study attitudes towards science include interviews (Bray and Ankeny, 2017), focus groups (Macnaghten and Guivant, 2011), case studies (Shaw, 2002; Williams et al., 2017) and the Draw-A-Scientist-Test (Newton and Newton, 1992; Tan et al., 2017). Such studies have provided more in-depth understanding of attitudes towards science, but often their findings are highly contextualized and hard to generalize. Furthermore, these studies often are dedicated to a specific situation when negative attitudes towards science become apparent or even problematic, such as fracking (e.g. Williams et al., 2017) or genetically modified organisms (e.g. Shaw, 2002). As such, attention in these studies goes to negative attitudes or concerns, leaving little room for positive attitudes.

We opt for a novel approach that allows us to identify bottom-up current and rising attitudes towards science and analyse letters to the editors in two major Dutch newspapers. Science is not a dominant theme in these newspapers; they are national newspapers covering a broad range of aspects. The opinions and perceptions are expressed without interference of a researcher, and not in the context of research into attitudes towards science. This enables us to study the attitudes of the authors of these letters as a fly on the wall. It enables us to include a broad range of academic fields, from the (natural) sciences to the social sciences and humanities. Moreover, it allows for capturing both positive and negative attitudes. Most importantly, it allows for identifying attributes, circumstances and understandings of science that seem to influence attitudes towards it. A letter to the editor requires the author to not just formulate a pro or con statement, but also to substantiate this with arguments or reasoning. As such, we believe that this study on attitudes towards science provides insight beyond the state-of-art, which focuses mostly on attributes of social groups, and allows to explore attributes of science.

The remainder of this article is organized as follows. In section 2, we briefly discuss literature about attitudes towards science to position the contribution of our study, and we formulate a broad guiding research question. In section 3, we describe the methodology in more detail. We discuss the results in section 4. In section 5, we return to our research question and reflect on further research and practical implications.

## 2. Contribution of the study to existing literature

Before discussing the literature on attitudes towards science, we define ‘attitudes’. We adopt the definition coined by Osborne et al. (2003) and understand attitudes towards science as ‘the feelings, beliefs and values held about an object that may be the enterprise of science, school science, the impact of science on society or scientists themselves’.

The majority of studies originate in the United States and countries across Europe. A major insight from studies comparing attitudes in different countries is the post-industrialism effect. This entails that publics in both the least and the most industrialized countries are more likely to hold negative attitudes towards science. In post-industrial countries, a knowledgeable disinterest has been put forward as an explanation for negative attitudes in the most industrialized countries (Bauer et al., 1994).

Yet, the results of most studies indicate that attitudes towards science are very positive in modern Western societies, especially when compared with other institutions, such as government, parliament, the press or jurisdiction (House of Lords/Science and Technology Committee, 2010; Miller, 2001; Millstone and van Zwanenberg, 2000; Tiemeijer and De Jonge, 2013). In the United States, this attitude is less positive than in Europe. Nevertheless, the attitude towards science is still relatively positive; the military is the only institution that receives a higher score than science (National Science Board, 2018).

Despite this general positive attitude, some people hold a positive attitude towards scientific methods and principles, and at the same time hold a negative attitude towards, or are concerned about, scientific organizations. Achterberg et al. (2017) associate this ‘confidence gap’ with education level. They find a larger gap among the lower educated and a smaller gap among the higher educated. The explanation they offer is anomie; a feeling of cultural malaise and not-belonging, of being threatened by impersonal modern institutions such as science. Yet, at the same time, science understood as clear methods, can play an important role in restoring a meaningful order.

Comparable with Achterberg et al. (2017), the vast majority of studies on attitudes towards science seem to focus on identifying attributes of social groups. Educational background (Bak, 2001; Guenther and Weingart, 2016), gender (Luján and Todt, 2000), religion (DiMaggio et al., 2018; McPhetres and Zuckerman, 2018) and ideology (Pechar et al., 2018) have been found to play a role in shaping attitudes towards science.

Some studies are dedicated to attitudes towards a specific research field or topic, often either emerging technologies or controversial fields. For instance, Dijkstra and Schuijff (2016) reviewed the literature on human enhancement technologies and conclude that the majority of lay people hold negative attitudes towards these technologies. Young (2013) studied letters to the editor about climate research and science in Canadian newspapers. He concludes that, despite scientific consensus, climate science regularly is contested in these letters. Shaw (2002) interviewed lay people in the UK to understand their attitudes towards genetically modified food. She found that scientists were distrusted, a sign of a negative attitude. However, these studies focusing on specific fields, including on contested cases, do not present much evidence of attributes of science that are of influence on the attitudes towards it. Finally, more established, less controversial fields and especially the social sciences and humanities can count on very little coverage in the literature.

Gauchat (2012) seems to be an exception regarding the dominant focus on attributes of social groups. He writes about attributes of science that may explain differences in public attitudes towards it. He aimed to test, by means of a survey, whether conservatives in the United States have increasingly developed negative attitudes towards science. To explain attitudes towards science, he points at generic perceptions, in the sense of what science is understood to be. He identifies three different perceptions: (a) science refers to a systematic method; (b) science is done or localized in certain institutions, such as a university and (c) science relates to specific knowledge that should accord with common sense and tradition.

Thus, there is a plethora of studies that investigate the attributes of social groups to explain their attitude towards science. Despite differences in the attitudes of these groups, the attitude can be considered quite positive in general. Then how do we explain the controversies science is faced with? Some of the studies reviewed above (Dijkstra and Schuijff, 2016; Gauchat, 2012; Pechar et al., 2018; Shaw, 2002; Young, 2013) provide indications that attributes of science, such as the research topic, may influence the attitudes towards science. Yet, knowledge on these attributes is rather limited. Therefore, the main research question that guides our explorative study is ‘What perceived attributes of science contribute to attitudes toward science?’ To answer this question, we must also investigate what people say about science, rather than merely establishing their attitude. This demands a qualitative approach, by means of content analysis, which we elaborate on in the following section.

## 3. Methodology

Previous research indicates that attitudes towards science are multi-faceted and need a more detailed exploration. To move beyond existing theoretical frameworks, we opt for the inductive approach of interpretative content analysis (Bray and Ankeny, 2017). This allows the data to reveal the various positive and negative attitudes towards science. Similar to Young (2013) and Silva and Lowe (2015), who analysed the debates on climate change and evolutionary theory, respectively, we use letters to the editor. This allows us to capture freely expressed thoughts, opinions and experiences. Also, it facilitates identifying bottom-up emerging themes. The major advantage compared with surveys and interviews is that there is no interference by a researcher. The expectation is that analysing this data source using content analysis opens the way for new theoretical explanations (Gioia et al., 2012). The Netherlands provides an interesting case as it closely resembles the European average concerning attitudes towards science as measured through the Eurobarometer (European Commission, 2010).

### Data collection

Letters were collected from two national Dutch dailies that we assume to capture both ends of the spectrum of attitudes towards science in the Netherlands. The first is ‘De Telegraaf’, generally acknowledged as leaning towards a populist signature, having the largest circulation in the Netherlands (National Onderzoek Multimedia, n.d.). ‘De Telegraaf is close to its readers and tells in clear language what is happening in their world, in the Netherlands and the rest of the world’.^1^ Generally, its readers have lower levels of education and income. The second is *NRC Handelsblad*, a quality, or even elitist, newspaper having the fourth highest circulation (National Onderzoek Multimedia, n.d.). ‘With quality journalism, we focus on the decision maker of today and tomorrow’.^2^ On average, its readers have the highest levels of education and income of all Dutch newspapers.

We used LexisNexis to retrieve all letters to the editor discussing science published by these two newspapers between 1 January 2007 and 5 June 2012.^3^ We searched on the name of the respective titles of the letters to the editor sections in combination with ‘wetenschap’; the Dutch word that, comparable with the German word ‘Wissenschaft’, refers to the sciences as well as the social sciences and humanities.

### Data description

The search resulted in a final set of 302 letters. Of these letters, 72 were published in *De Telegraaf* and 230 in *NRC Handelsblad*. Although *De Telegraaf* published significantly less letters than *NRC Handelsblad*, the result of our search shows that science is an issue to readers of both newspapers, nuancing earlier findings of ‘knowledgeable disinterest’ in the most industrialized countries (Bauer et al., 1994).

Based on the content of the letters and information provided about the author, we classified the letters into three categories. The first category includes 107 letters written by representatives of the science system. It includes letters written by people who sign their letter with professor, doctor and/or who state an affiliation to a university in their letter, as for example, the head of libraries of the University of Amsterdam did. These representatives have in-depth insights in science. The second category comprises letters written by experts, either by profession or by experience, on the topic of the letter. We created a specific category for experts as a study by Retzbach et al. (2011) suggests that lay people who are more engaged with science tend to have different attitudes from those who are not. Examples are a psychologist working in healthcare writing about psychological research and a director of a renewable energy company writing about sustainability issues. This category includes 36 letters. The third category is letters by lay people. A letter was assigned to this category if neither from references in the letter, nor from the signature we could deduce a relation to the topic of the letter, neither as an expert, nor as a representative of the science system. This category includes 159 letters. A breakdown of letters per author category per newspaper can be found in Table 1.

**Table 1.** Breakdown of letters per newspaper and author category.

	Representative of science system	Expert	Lay person	Total
*De Telegraaf*	2	2	68	72
*NRC Handelsblad*	105	34	91	230
Total	107	36	159	302

### Data analysis

Two members of the project team (S.P.L.d.J. and L.K.) analysed the letters in an iterative process using coding Software Atlas.ti. The analysis consisted of three steps: open coding, axial coding and selective coding (Strauss, 1987; Strauss and Corbin, 1998). Open coding was used to identify bottom-up emerging first-order concepts. Then, all three members were involved in axial coding. They discussed the results and jointly clustered codes into three main themes, each consisting of two sub-themes. Letters as well as quotes might cover more than one theme and sub-theme per theme. Finally, selective coding was done using the six sub-themes. Not all letters could be allocated to one of the themes. These remaining letters cover a large variety of topics, often only including a small number of letters per topic. See Table 2 for these themes and sub-themes, as well as for examples of the underlying first-order concepts, inspired by Gioia et al. (2012).

**Table 2.** Data structure.

First-order concepts	Sub-theme	Theme	Total number of letters	Representative of science system (% of total letters per theme)	Expert (% of total letters per theme)	Lay person (% of total letters per theme)
Autonomy	Proper science	Enlightenment ideals	104	33 (32%)	14 (13%)	57 (55%)
Verifiability						
Method						
Peer review						
Truth-finding						
Trust in scientists	Trust in science					
Societal progress						
Science used to support an argument						
Science as independent critic						
Reputation of organization	Organizational culture	Organization	20	16 (80%)	0 (0%)	4 (20%)
Financial interests						
Governance						
Emphasis on positive results	Scientific culture					
Quantification						
Private sector	Conflicts of interest with non-academic institutions	Conflicts of interest	50	19 (38%)	5 (10%)	26 (52%)
Politics						
Media						
Patients						
Usefulness						
Career progression	Conflicts of interest with individual academics					

Quotes are translated from Dutch to English. Square brackets indicate editing by the project team or clarification of the Dutch context. We have translated ‘wetenschap’ to ‘science’ to present quotes as close as possible to the original Dutch version, despite the more narrow definition of science in English (see section ‘Data collection’). The first letter between round brackets following each quote refers to the newspaper the quote is from (‘N’ for *NRC Handelsblad* and ‘T’ for *Telegraaf*). The second letter refers to the type of author (‘S’ for representative of the science system, ‘E’ for expert and ‘L’ for lay person.) We use ‘authors’ when we refer to those readers who wrote a letter to the editor. When we use ‘readers’, we refer to the full audience of the newspaper.

## 4. Results

We have identified three major themes in the letters. The first regards perceptions and expectations of science. These letters address what science is, they draw a line between science and non-science, and identify science as a necessary source of knowledge. They comprise, in general, positive attitudes towards science. The second is ‘organization’, which is about the influence of market thinking on universities and researchers. These letters address governance and management of scientific organizations and the consequential behaviour of scientists. The third is ‘conflict of interest’ and is about private, public and individual motives that impair science. These letters address the use of scientific results by third parties and scientists looking for personal gain. The latter themes reflect negative attitudes that can be understood to originate in certain attributes of present-day science. Table 2 shows the underlying first-order concepts per sub-theme, the total number of letters per theme and the number of letters per author category per theme.

### Proper science as a reliable and necessary source

Several authors define science and demarcate science from non-science. The basic perception seems to be rooted in Enlightenment ideals: science as pure and inherently good. And that makes science a trustworthy source of knowledge, needed to support societal progress. We identify two sub-themes. The first addresses what science is, it defines proper science and identifies claims that cannot be considered scientific. The second addresses science as a source of knowledge that can be trusted to support societal progress, and that should be used to do so. Enlightenment ideals are primarily discussed by lay persons, to a lesser extent by representatives of the science system and occasionally by experts.

To start with the sub-theme of proper science, according to the letters, scientific research is a specific activity that is done according to certain rules. Several authors formulate these rules. Science is objective, verifiable and done according to Karl Popper’s rules, as the following quotes from *NRC Handelsblad* show: ‘In my opinion, in scientific research, verifiability, let’s say provability, is provided by the fact that the presented data can be “checked” for the largest possible level of truth by a third party’ (N/L) and

Usually, from the reporting, I cannot deduce whether a sound and credible hypothesis has been formulated – hence, a thesis that states the opposite of what one hopes to find and that should be falsifiable based on the data in order to be allowed to accept your own thesis (my paraphrase of how Sir Karl Popper taught science to behave). (N/L)

Furthermore, the application of such rules and methods distinguishes science from non-science. Multiple authors say, it is not science, if the rules are not applied, as the following quotes exemplify: ‘That term [“we”] is not scientifically defined (from the perspective of whatever discipline) by Swaab [a professor of neurobiology and author of popularizing books]. In daily life that is not an insurmountable problem, but in science it is’ (N/E) and ‘There is no analysis and the presented solution is based on the “I believe” method. That is not scientific’ (N/S).

These criteria do not relate to (natural) sciences only, they relate to humanities research as well. Yet, an extra criterion is added: sophisticated arguments. ‘A historian contributes to increasing the knowledge pool with his scientific research. For him, there is no higher goal than the research results themselves. Scientific criteria apply to his research: objectivity, verifiability and high-quality arguments’ (N/L).

Also, science is the exclusive domain of academics, and there is a clear division of labour between academics and non-academics. Non-scientists might be involved in application of research results, but not in the scientific research process, as an author argues,

In science, hypotheses are formulated, tested and commented on among scientists. Non-scientists do not have a voice in that debate, but they do when it is about applications in society. Often, this distinction is forgotten about, assuming that everyone always is allowed to join a discussion. (N/S)

Now that science has been defined as a method with strict rules and principles, done by special people with no other interest than science itself, and demarcated from non-science and non-scientists, who should not participate in science, the question is: why is science important? This brings us to the second sub-theme. From the letters, the image emerges of science as a trustworthy source of knowledge, due to the rules and principles. And since it is trustworthy, it must be true. Authors mention a specific attribute that makes science especially trustworthy: its expected independence. Science and its independence are often used to strengthen an argument following the general format of ‘according to an independent scientist, thus true’. The following phrases illustrate the use of this format: ‘Recent research by independent researchers from all over the world [. . .]’ (N/E) and ‘Both facts support the thesis of independent scientists’ (T/L). Some even go as far as to stress the ‘uselessness’ of science (humanities in this case) as an important quality of science: ‘The humanities are useless in economic terms. Moreover, they are a thorn in one’s side, being the only faculty with an independent and critical position regarding the private sector’ (N/L).

However, there are limits to uselessness. Some authors have doubts when researchers spend time on, in their eyes, ridiculous topics. One such example:

On 10 July 1585, William of Orange [‘father’ of the Dutch nation] was killed by a number of gun shots. At present day, March 2009, scientists came up with the ridiculous idea of studying the precise circumstances of the murder. [. . .]. All scientific idiocy. Complete foolishness. (T/L)

The consequence of choosing the wrong topics remains unnamed in the letters. But if and when the right topics are chosen, scientists and scientific evidence are considered to be reliable, trustworthy sources of knowledge that one should respect. Several authors make this point. Two examples, one addressing the scientist as source, the other scientific evidence, include: ‘Van de Vraats is a professor of mathematics in Amsterdam and he knows what he is talking about’ (N/S) and ‘To label these disorders as brain disorders demonstrates not only misguidance, but also a serious lack of respect for scientific insights’ (N/S).

The importance of science as a reliable knowledge source becomes even more apparent, when it is absent. One should not accept just anything without clear scientific proof: ‘This lack of criticism leads to a society in which all kinds of statements are accepted as true, without any scientific justification’ (N/L). And to continue in that line, authors advise to use, and thus not to ignore or to mistrust, scientific knowledge, for example: ‘To prevent returning to the situation before the year 1300 I propose to use available knowledge from the natural sciences from now on when predicting the weather and climate’ (N/L). Indeed, scientific research and scientists have provided very useful insights, much needed outside academia as the following quotes indicate: ‘Now that there is more research on sustainable agriculture, the lower yield issue will soon be addressed’ (N/E) and ‘According to oncologists dr. Kostler in Vienna and dr. Douwes in Bad Aibling (Germany), these deodorants should no longer be sold as “they are a threat to our general health”’ (T/L). Notice that the (future) results are to the benefit of all.

In other cases, where scientists have not contributed yet, authors ask scientists to take action. They must come to the rescue: ‘We don’t hear much from philosophers in this debate, although their contribution could be clarifying’ (N/E) and ‘Perhaps a historian of war could provide an decisive answer on this matter’ (T/L).

Also, according to the authors of the letters, it is wise to invest in science, because science helps mankind: ‘Thanks to science, we are about to enter a new era’ (T/L).

So, from the letters, we learn that when research is done according to certain principles and done by certain people, it deserves the label ‘proper science’. If these scientists are independent and working on the right topics, science is a trustworthy and much needed source for societal progress according to the authors. However, the authors also discuss some serious threats.

### Business-like management

The first threat authors perceive relates to the organization of science. The first sub-theme is organizational culture, which relates to the governance and management of science and universities, or rather the non-scientific and business-like management style. The second is academic culture, which relates to the behaviour of scientists. The two seem to be linked to each other, as the former can be perceived as affecting the latter. Again, Table 2 provides an overview of the first-order concepts behind the sub-themes. Organization of science is a theme that mainly representatives of the science system write about as well as some lay persons. This theme was not represented in letters by authors from the expert category.

Authors observe that universities are being led by managers, who are at a distance from the actual research process and who are too close to policy:

Nowadays, the dean is a subordinate to the board. That is where policy is being made, by people who can’t possibly have in-depth knowledge on research and teaching across the full spectrum. (N/S)

These managers are accused of focusing too much on use and the possibility to earn from research:

The medical centre of the Free University Amsterdam is, comparable to other academic institutions in the Netherlands, governed in line with the neoliberal philosophy of the current government: knowledge production and scientific research should be useful for the Netherlands and have economic returns, otherwise they can be dissolved. (N/S)

The researchers are affected by this governance philosophy. They spend a considerable amount of time attracting funding, time that cannot be spent doing proper research: ‘According to the researchers in the article, professors are increasingly unavailable on the work floor, as they are focusing on attracting funding’ (N/S). Moreover, authors fear that this type of finance, that already takes too much time to secure, does not stimulate basic research:

Because the battle for big money for truly fundamental research often is less successful than having employees in unscientific projects that support our private sector, the latter increasingly is the case. (N/S)

On top of that, authors identify that as a consequence of the current governance there is too much focus on: ‘quantity and not [. . .] quality’ (N/L and N/S). Authors are concerned about the consequences of this focus on quantity, in particular on published articles. They mention resulting ‘perverse incentives’ (N/S) and the preference of journals to publish positive results only: ‘And in science, there are indeed incentives to show certain results. Positive results are more easily published’ (N/S). Authors mention that scientists are forced to publish ‘more’ rather than ‘good’ (N/S) and ‘Tend to publish mainly successes’ (N/S). And just to be sure, these observations are indeed linked to university management: ‘Scientists have to publish for the prestige of their university’ (N/S).

In short, the business-like management culture leads to too much emphasis on money, use and publications. This affects the behaviour of scientists and is at the expense of (the principles of) basic research. The ultimate consequence is fraud. ‘If NWO [Netherlands Organisation for Scientific Research (Dutch research council)] only selects “excellent” researchers [. . .] based on the number of publications, than the odds are that NWO relatively attracts many frauds’ (N/S). This leads to a different type of concern: conflicts of interest.

### Conflicts of interest

Authors mention two concerns that regard conflicts of interest. One relates to a specific circumstance that is perceived as very unfavourable: any connection with and use of scientific results by non-academic organizations. One of the dangers is that whoever pays, determines the results, and this might tempt scientists to manipulate data. And it might put scientists on a slippery slope, leading to the other conflict of interest: personal gain. Fraud, as previously mentioned, is one illustration of this conflict. Table 2 lists the supporting first-order concepts per sub-theme. Just over half of the letters that feed into this category could be assigned to lay people, over a third is written by representatives of the science system and only a small number of letters on this theme could be assigned to the expert category.

When it comes to connections with and use by non-academic organizations, many authors formulate concerns. In general, the authors see the private sector as evil: ‘Is there any faculty which has not sold her soul to the private sector in the past decades?’ [N/L], and they accuse the private sector of influencing scientific results: ‘Too often the client determines’ (T/L). The rules of proper science, that determines the value of science, are being denied in this particular context: ‘the scientific habit to attempt to falsify hypotheses is often seen as an annoying obstacle [. . .] for useful application of knowledge’ (N/S).

Several authors refer to medical science and the relation to ‘the corrupt pharmaceutical industry’ (N/L) and ‘Mainstream medicine is privatised and commercialised’ (T/L).

Others refer to the field of agriculture and accuse researchers of neglecting public values:

Because we humans don’t react well to growth hormones [for poultry], scientists once more invented something, which can be added to chicken feed, so that we can eat chicken without any worries again. Of course, we will find out that these chicken suffer from side effects of all these remedies, but I’m sure they will be able to invent another pill for that. (T/L)

Authors have doubts when non-academic organizations use results from academic research, and this is not restricted to the private sector: ‘Pension funds use every opportunity to lower their payments. This time they found a scientist who has figured out that we will live even longer’ (T/L) and ‘I wonder which friendly ministry commissioned this “scientific report” and what it paid for that’ (T/L). Yet scientists are not without blame either, they manipulate data, for instance for a lobby that is certainly not organized by the private sector: ‘Scientists manipulate figures about global warming in favour of the environmental lobby’ (T/L).

Also, scientists present results that are too good to be true: results that are viewed with scepticism by the authors:

Around New Year’s Eve we learned that according to scientists ‘oliebollen’ [Dutch donuts, traditionally eaten during New Year’s Eve] are healthier than ‘eierkoeken’ [Dutch sponge cakes, promoted by a Dutch health guru]. And now, a week before Easter, when chocolate eggs are being sold in huge quantities, I’m reading that scientists proved that you lose weight by eating chocolate. (T/L)

Authors seem to understand how such studies come about, and again are sceptical. It is the individual researcher who knows how to play the game, who wins: ‘Collier belongs to the international elite of economists, who, as academics and advisers to the one or the other power, constantly try to dethrone each other, at the expense of the people it should be really about’ (N/E) and ‘In my opinion, these learned gentlemen are after bonuses for historical discoveries’ (T/L).

Also, authors are not afraid to name several forms of ‘minor criminal offenses’ (N/S), related to such personal gain: ‘plagiarism’ (N/S), ‘hunting for statistical significance’ (N/S) as well as ‘abuse of copyright’ (N/S).

Meanwhile, the independent, critical and honest truth-seekers are declining in numbers:

Furthermore, there was a scientist from the Academic Medical Centre in Amsterdam in the TV-show who believed the fuss about radiation was much ado about nothing and who dared to give an opinion that was not politically correct. My experience is that nowadays this is very rare among scientists who work at universities. (T/L)

Comparable with worries about the organization of science, the authors expect interests of non-academic organizations and individual academics to affect proper science as a trustworthy source of societal progress.

## 5. Concluding remarks

### Conclusion and discussion

The start for our analysis was the question ‘What perceived attributes of science contribute to attitudes toward science?’

We identify a strong image of science. Science, we learn from the letters, is done according to certain rules. Thus, science is defined as a specific method. Examples of such rules are objectivity and verifiability. Following these rules makes science trustworthy and results in a positive attitude. Also, science is the exclusive domain of certain people that work in universities which are independent from other organizations. These findings strongly resemble the first two perceptions of science that Gauchat (2012) identified: science as a systematic method and science as localized in certain institutions, such as a university. According to the authors of the letters, universities should safeguard the circumstances that are required to work according to scientific method and rules. If they succeed in this task, one can and should trust science and scientists, since they provide the independent and objective knowledge, analyses and expertise required for societal progress.

Negative attitudes towards science, or even distrust, root in not adhering to these rules as defined by a particular individual or societal group. The authors of the letters identify two reasons for science and scientists to break the rules. The first relates to the organization of science. In the perception of the authors of the letters, market or business thinking within universities and (induced) competition among scientists, incentivizes scientists to adopt norms that contradict traditional scientific norms. Universities are perceived as no longer safeguarding the scientific method. The second is conflicts of interest from public and private entities with too much influence on science, as well as from scientists who lose the scientific principles out of sight when focusing too much on personal profits and gain. The demarcation of who is allowed to be involved in scientific research is not respected and as a result the scientific method is impaired. In other words, it is not necessarily science or scientists (Shaw, 2002) that are mistrusted, but rather the institutions that are supposed to safeguard it/them or that it/they collaborate(s) with.

The attributes of science that we have found to affect attitudes towards it closely resemble Merton’s (1973: 270–278) observations concerning the normative structure of science. According to Merton, ‘science’ can refer to the following:

(1) A set of characteristic methods by means of which knowledge is certified; (2) a stock of accumulated knowledge stemming from the application of these methods; (3) a set of cultural values and mores governing the activities termed science; or (4) any combination of the foregoing.

In particular, we recognize (1) and (3) in the letters to the editor. They resemble the findings of Gauchat (2012), but notice that Merton mentions (3) the set of values and norms that govern science, whereas Gauchat merely identifies the organizations as such. Merton also defines four imperatives that guide scientists. These are communism (common ownership of knowledge), universalism (all scientific claims should be assessed using the same criteria, which should not concern personal or social attributes of the individual presenting the claim), disinterestedness (scientists should not work for personal gain but for the common scientific good) and organized scepticism (scientific claims should not be accepted unless critically assessed.) Again, these norms, referred to as CUDOS, are reflected in the data, disinterestedness and scepticism perhaps most visibly. Finally, comparable with Merton (1973: xix) as well, authors of the letters pose that when non-scientific norms are introduced to science, such as management or commercial norms, scientists are more likely to disrespect scientific norms, which may even lead to fraud. As we expect few to none of the authors to have a background in sociology of science, this resemblance is quite remarkable.

Although not part of the main analysis, we found some interesting differences in the relative prominence of the themes in the two newspapers. Enlightenment ideals and conflicts of interest are more often discussed in letters from *De Telegraaf*. Given the profile of the readers of *De Telegraaf* (lower educated and potentially higher levels of anomie as compared with those of *NRC Handelsblad*), this comes as no surprise. It can be understood as a positivistic image of science (Enlightenment) and a general lack of trust in intentions of modern institutions (conflict of interest). As such, it resonates the findings of Achterberg et al. (2017) concerning the science confidence gap, which is larger among the lower educated. The negative effects of science’s organization is more often discussed in *NRC Handelsblad*. Given that the vast majority of letters authored by representatives of the science system is published in *NRC Handelsblad*, again this is hardly a surprise. This is also reflected by the vast majority of the letters on this theme that were written by authors from this category, whereas lay persons are far less represented in this theme compared with the other two themes. The worries of representatives of the science system may be part of the explanation for the cautiousness among groups with a high scientific literacy (Guenther and Weingart, 2016). Yet, literature seems to position the sentiments regarding the organization of science as an internal matter, related to changing management of universities (e.g. Lorenz, 2012; Teelken, 2012), rather than as a public concern.

So, positive attitudes towards science root in its methods, its actors and the societal contributions it can make. Negative attitudes, in our sample, can be explained by the organization of science and conflicts of interest that move science and scientists away from scientific procedures and norms, and away from contributing fundamental knowledge that in the end will be used for meaningful societal goals. In addition to factors that relate to attributes of individuals and social groups, as identified by earlier studies, our findings show that attitudes towards science are shaped by attributes of science, scientific institutions and scientists as well.

### Methodological reflections

Choosing newspapers as a data source to tap into people’s thoughts on science turned out to be productive. The retrieval of over 300 letters suggests that newspapers are a forum to discuss science, both in terms of its results as well as a social phenomenon. Lay persons, experts on specific topics and scientists and other representatives of the science system alike participate in the discussion. Yet, the discussion is more lively, as indicated by the higher number of letters and the diversity in types of authors, in *NRC Handelsblad* compared with *De Telegraaf*.

When generalizing our results, it should be kept in mind that selecting the Netherlands as a case may have affected our results. A number of academic fraud cases took place in the Netherlands during the time window for data collection. The Stapel affair is probably the most well-known – Stapel, a prominent professor in social psychology, was found guilty of fabricating data on a large scale (Enserink, 2012). The societal debate on academic fraud at the time may have resulted in a higher number of letters about scientific culture and conflicts with interests of individual academics as would have been the case without these cases.

Also, neoliberalism^4^ has a strong influence on the organization of science in the Netherlands (Seeber et al., 2015). This could explain why we find the organization of science to be a root for distrust. In countries in which neoliberalism is less influential, the organization of science might not be such a prominent root of distrust.

### Recommendations for further research and practice

Our study demonstrates that attributes of science influence attitudes towards it. Follow-up research could consider what attributes are most influential and whether there are differences among social groups regarding science’s attributes that affect their attitudes towards science. The outcomes of these studies could support interventions aiming to promote positive attitudes. We anticipate that such interventions will not merely focus on communicating and even glorifying scientific results, but rather on processes and how scientific norms are respected and protected.

In addition, our study identified a clear public expectation that science contributes to progress, yet, such contributions may not be produced in collaboration with society, in particular industry and government. This tension requires future research as well as efforts in practice, as research has shown that collaboration between science and society is conducive to or even a requirement for societal progress (Etzkowitz and Leydesdorff, 2000; Funtowicz and Ravetz, 1993; Gibbons et al., 1994). On a more practical level, governments and research funders increasingly require academics to collaborate with industry, or even require co-funding, when allocating research funds. Given the concerns about the involvement of industry, such (neoliberal) measures to increase societal benefits of science may backlash and negatively affect the societal acceptance of the results from these collaborations. Future research should further unravel why such collaborations are perceived to be problematic and how public concerns can be mitigated.

Our results also serve scientists and scholars wishing to participate in public debates about science, such as in newspapers. Our study confirms that newspapers are a forum for debates about science. However, in our sample, scientists hardly participated in the debate in the populist newspaper. Yet, science is being discussed in this newspaper, suggesting that its readers do have an interest in science. As such, scientists should consider joining the debate in a larger variety of newspapers to connect to different types of audiences. A first suggestion for a topic to discuss is how the involvement of non-academic institutions, if well regulated, can facilitate research that contributes to societal progress.

## Notes

1. https://www.werkenbijtmg.nl/onze-merken/709-de-telegraaf (translation by the authors).

2. https://www.nrcmedia.nl/geschiedenis-nrc/ (translation by the authors).

3. The date of data collection in LexisNexis.

4. A political philosophy aiming at a more efficient organization of the public sector by introducing market mechanisms into this sector (Pollitt and Bouckaert, 2004).
